# Advancing Neurosurgical Oncology and AI Innovations in Latin American Brain Cancer Care: Insights from a Center of Excellence

**DOI:** 10.3390/neurosci6020054

**Published:** 2025-06-10

**Authors:** José E. Valerio, Immanuel O. Olarinde, Guillermo de Jesus Aguirre Vera, Jorge Zumaeta, Noe Santiago Rea, Maria P. Fernandez Gomez, Penelope Mantilla-Farfan, Andrés M. Alvarez-Pinzon

**Affiliations:** 1 Neurosurgery Oncology, Latinoamerica Valerio Foundation, Weston, FL 33326, USA; jevalerio@jvaleriomd.com (J.E.V.);; 2 Department of Neurological Surgery, Palmetto General Hospital, Miami, FL 33016, USA; 3 Neurosurgery Oncology Center of Excellence, Department of Neurosurgery, Miami Neuroscience Center at Larkin, South Miami, FL 33143, USA; 4 GW School of Business, George Washington University, Washington, DC 20052, USA; 5 Instituto Tecnológico y de Estudios Superiores de Monterrey (ITESM), School of Medicine, Monterrey 64700, Mexico; 6 The Institute of Neuroscience of Castilla y León (INCYL), Cancer Neuroscience, University of Salamanca (USAL), 37007 Salamanca, Spain; 7 Hospital Real de Granada, Universidad de Granada, 18014 Granada, Spain; 8 Institute for Human Health and Disease Intervention (I-HEALTH), Florida Atlantic University, Jupiter, FL 33433, USA; 9 Stanford LEAD Program, Stanford Graduate School of Business, Stanford University, Palo Alto, CA 94305, USA

**Keywords:** brain tumors, neuro-oncology, Latin America, health inequities, LMICs, health systems, neurosurgery

## Abstract

**Background:** Disparities in neuro-oncological care between high-income and low- and middle-income countries (LMICs) are well documented, yet region-specific data from Latin America remain limited. This review evaluates epidemiologic trends, access to care, and systemic challenges in brain tumor management across Latin American LMICs, using Argentina as a case study. **Methods:** A systematic review of peer-reviewed literature was conducted focusing on brain tumor incidence, mortality, risk factors, and availability of diagnostics and treatments in Latin America. Socioeconomic, cultural, and systemic barriers were also analyzed. **Results:** Latin America exhibits some of the highest global brain tumor mortality rates, with Brazil reporting age-standardized rates exceeding 4.5 per 100,000. Glioblastomas are frequently diagnosed at younger ages, often in the fifth decade of life, compared to the global average. Meningioma incidence has increased by 15–20% over the last decade, yet region-wide data remain fragmented. Access to neuroimaging, neurosurgery, radiotherapy, and chemotherapy is limited, with up to 60% of patients relying solely on under-resourced public health systems. Less than 30% of hospitals in rural areas have MRI availability, and continuous professional training is infrequent. Innovative adaptations, such as awake craniotomy, are used in some LMIC centers in response to equipment scarcity. **Conclusions:** Brain tumor care in Latin America is hindered by limited epidemiological data, restricted access to diagnostics and treatment, and insufficient workforce training. Targeted investments in healthcare infrastructure, international educational collaborations, and policy-level reforms are critical to reducing disparities and improving outcomes in neuro-oncology across the region.

## 1. Introduction

Recent developments in cancer neuroscience have elucidated the complex interactions between the nervous system and cancer, revealing a bidirectional relationship that has a substantial impact on both patient outcomes and the progression of the disease [1]. It is acknowledged that the nervous system, which includes both central and peripheral components, is responsible for regulating critical processes, including development, homeostasis, plasticity, and immune function, as well as the initiation and progression of cancer [2]. The development of cancer may be influenced by disruptions to these physiological processes, whether through alterations in neuronal signaling, immune response modulation, or changes in the tumor microenvironment, according to emerging evidence. In contrast, cancer and its treatments can trigger a series of neurophysiological changes that result in neurological dysfunction, thereby establishing a feedback cycle that exacerbates tumor progression and complicates therapeutic interventions [3].

The molecular and cellular mechanisms that underline the interactions between cancer and the nervous system are the primary focus of this burgeoning field, which has given rise to cancer neuroscience. Researchers are investigating the potential for tumors to commandeer neurodevelopmental pathways for malignancy, as well as the potential for the nervous system’s response to cancer to enhance tumor survival, invasiveness, and resistance to drug treatment [4]. As a result, novel therapeutic strategies are being developed to address these interactions and to improve the outcomes for cancer treatment and neurological health [5]. Nevertheless, there are still numerous obstacles to overcome, particularly in the development of strategies that can effectively target these intricate neuro-cancer pathways without causing unintended neurological injury [6].

Simultaneously, healthcare disparities—particularly in low- and middle-income countries (LMICs)—continue to impede the availability of timely, effective cancer care, which includes the treatment of brain tumors. The healthcare infrastructure for cancer care in LMICs is still insufficient, particularly in the field of neurosurgery. A combination of limited healthcare access, inadequate surgical capacity, and shortages of specialized personnel means that many patients in these regions experience lengthy delays in receiving diagnosis and treatment [7]. In certain LMICs, patients are required to travel considerable distances to obtain surgical care, which results in the postponement of essential interventions and the worsening of patient outcomes. Despite the presence of neurosurgical centers in urban areas, there is frequently a dearth of critical post-surgical radiation and chemotherapy services [8]. Additionally, the ratio of neurosurgeons to the population in LMICs is alarmingly low, with some countries reporting the ratio as low as one neurosurgeon per 1 to 1.4 million individuals. These deficiencies pose substantial obstacles to the management of intricate conditions, including brain tumors, which necessitate specialized, multidisciplinary care [9,10,11].

To rectify these disparities, the World Federation of Neurosurgical Societies (WFNS) has formulated a classification system that evaluates the quality and accessibility of neurosurgical services in various cities. This system categorizes cities into four levels, which range from Level 0, which lacks neurosurgical services, to Level 3, which offers sophisticated microneurosurgery [12,13,14,15]. The availability of services is substantially reduced as the complexity of the necessary procedures increases. For example, in Latin America and the Caribbean, only 50.3% of the population has access to Level 2 neurosurgical services, and a substantial portion of the population must travel up to two hours to reach a center that provides these services [14]. Access to Level 3 facilities is even more restricted, affecting only 27.4% of the population. The fact that access to both public and private healthcare facilities varies greatly across the region further exacerbates these challenges. Only 55.9% of the population has access to a public facility within a two-hour travel radius, while 48.7% can access private facilities within the same timeframe [15,16,17,18,19,20,21].

This study aims to examine the epidemiology, risk factors, and treatment modalities of brain tumors in low- and middle-income countries (LMICs), with a specific focus on Latin America. Additionally, the paper explores strategies to mitigate healthcare disparities between high-income countries and LMICs. Proposed interventions include the expansion of telemedicine services, deployment of mobile neurosurgical units, increased training and retention of neurosurgeons, and the development of collaborative partnerships between academic institutions and regional healthcare systems. Addressing these disparities is essential to improving timely access to neuro-oncological care and enhancing survival outcomes among underserved populations [17].

## 2. Brain Cancer Risk Factors

The pathogenesis of brain cancer is a critical area of research, with a variety of potential risk factors being investigated. Somatic mutations in critical genes, including TP53, IDH1, and EGFR, are frequently detected in gliomas and other CNS malignancies, making genetic factors one of the most extensively researched. The significance of inherited genetic mutations in specific subgroups of patients is further underscored by the predisposition of individuals to develop brain tumors by genetic syndromes such as neurofibromatosis, Li–Fraumeni syndrome, and Turcot syndrome. However, the increasing prevalence of brain malignancies cannot be entirely attributed to genetic risk factors, which has led to a greater emphasis on environmental and lifestyle factors [18,19,20,21,22].

Among the environmental risk factors relevant to brain cancer epidemiology in Latin America, exposure to ionizing radiation represents a significant concern, especially in underserved populations with limited healthcare resources. Ionizing radiation continues to be one of the most strongly associated environmental risk factors for brain cancer, particularly in minors. It is well established that radiotherapy, which is employed in the treatment of other malignancies, is a risk factor for secondary brain tumors, particularly gliomas and meningiomas [19,23]. The mechanism of radiation-induced brain cancer is likely to involve DNA damage, which results in genomic instability and mutations. Improvements in molecular profiling and imaging have facilitated the identification of individuals at a higher risk, thereby enabling the development of more personalized monitoring strategies for patients with a history of cranial radiation exposure [20,24]. Addressing radiation-related risks through improved diagnostic technologies and personalized monitoring strategies is therefore crucial to reducing healthcare disparities and enhancing outcomes in Latin American neuro-oncology.

The effects of non-ionizing radiation, which are emitted by mobile phones, Wi-Fi, and other sources of electromagnetic fields (EMFs), have been less conclusively demonstrated. In contrast, although in vitro studies have suggested that radiofrequency radiation may elicit oxidative stress and promote genomic instability in glial cells, large epidemiological studies have been unable to establish a consistent link to gliomas or meningiomas. The interaction between EMFs and neural tissue is still a subject of considerable uncertainty; however, ongoing research, particularly in the context of advanced AI-assisted imaging, may provide more detailed insights into this potential risk factor [21,22,23,24,25,26].

Additionally, certain viral infections prevalent in Latin American populations may contribute uniquely to the region’s brain tumor epidemiology, warranting focused research and targeted healthcare strategies. In the context of brain tumorigenesis, viral infections have also attracted attention. The role of human papillomavirus (HPV) and Epstein–Barr virus (EBV) in CNS malignancies has been the subject of research, with EBV being notably implicated in lymphoproliferative disorders and certain gliomas [22,27]. Viral-induced inflammation, immune evasion, and genomic alterations in host cells are among the mechanisms by which viruses may contribute to brain tumorigenesis. The utilization of AI-driven genomics platforms to examine viral DNA integration and host cell mutations could offer a more sophisticated comprehension of viral interactions in glioma genesis and other brain malignancies [23,24,25,26,27,28,29]. Given that many Latin American healthcare systems currently lack robust screening and diagnostic capabilities for virus-associated tumors, integrating AI-enhanced diagnostic tools may provide a valuable pathway toward addressing these disparities.

Endocrine factors have been investigated, with a particular emphasis on hormone replacement therapy (HRT) and hormonal contraceptives. Gliomas frequently exhibit estrogen and progesterone receptors, which implies that sex hormones may affect tumor behavior. Nevertheless, the literature contains a variety of conflicting data regarding the potential of oral contraceptives or HRT to either increase or decrease the risk of brain tumors. This is likely a result of the heterogeneity in tumor subtypes and hormonal receptor status. The refinement of our comprehension of these relationships may be facilitated by AI-assisted data mining in large clinical databases, which accounts for a variety of confounding factors and concentrates on specific molecular tumor profiles [24,30,31,32].

Several studies have investigated the correlation between body mass index (BMI), metabolic factors, and the risk of developing brain cancer. Some of these studies suggest that an elevated BMI is linked to an increased risk of glioma [25,33]. Metabolic dysregulation, such as insulin resistance or altered lipid metabolism, can affect the tumor microenvironment, influencing tumor growth and invasiveness. Furthermore, the analysis of metabolic profiles in glioblastoma multiforme (GBM) using AI has uncovered new pathways of tumor metabolism that could result in more precise interventions, such as the use of metabolic inhibitors in conjunction with conventional therapies [25,33,34,35,36,37,38].

Another emerging area of interest for brain cancer risk and management in Latin America relates to nutritional factors, particularly vitamin D deficiency, which is notably prevalent in several countries across the region. The importance of vitamin D in cancer biology is becoming more widely acknowledged, as low serum levels are associated with increased incidences of a variety of malignancies, such as gliomas [26]. Research indicates that vitamin D may affect the progression of tumors by promoting anti-inflammatory pathways and modulating immune responses. AI-assisted biomarker analysis in clinical trials has demonstrated that vitamin D signaling pathways may interact with critical tumor suppressor genes in glioma cells, thereby establishing a potential therapeutic target for cellular therapies [27,39]. Should future research confirm a direct causal link between vitamin D deficiency and increased brain tumor prevalence, addressing this deficiency through targeted nutritional interventions or supplementation programs could indeed become an accessible and cost-effective strategy to mitigate brain cancer risk and improve patient outcomes in underserved Latin American populations.

The early detection and treatment of brain cancer are also transformed by AI-based techniques. Modern machine learning algorithms are now capable of analyzing neuroimaging data to detect subtle changes in brain tissue, thereby providing early indications of a tumor presence before the onset of clinical symptoms [28,33,35,36,37,38,39,40]. AI algorithms can aid in personalizing treatment plans by identifying correlations between genetic and molecular data and patient outcomes. This facilitates a precision medicine approach to brain cancer care. Additionally, AI plays a crucial role in developing models that predict treatment responses and relapses, allowing for real-time adjustments to therapy regimens [29]. While AI-driven technologies significantly enhance the accuracy and personalization of brain cancer detection and therapeutic planning, their integration with novel cellular and gene-based therapies promises to further amplify treatment efficacy. Specifically, combining advanced machine learning insights with innovative approaches such as CAR-T cells and CRISPR gene editing may enable more precise targeting of aggressive tumors, optimizing outcomes and ushering in a new era of multidisciplinary neuro-oncological care.

Advancements in gene editing and immunotherapy are demonstrating potential in the treatment of brain cancer within the field of cellular therapies. The prospective strategy for treating aggressive gliomas, such as GBM, has been realized through the use of CAR-T cells, which are specifically engineered to cross the blood–brain barrier [30]. In the same vein, CRISPR-based gene editing technologies enable the direct targeting of oncogenic mutations in brain tumor cells, thereby offering a highly specific and efficient therapeutic approach. The efficacy of integrating these state-of-the-art cellular therapies with conventional neurosurgical interventions, including resection and radiotherapy, is currently being investigated in ongoing clinical trials to enhance the survival rates of brain cancer patients [31,37,38,39,40,41,42,43].

In summary, the intricate etiology of brain cancer is influenced by many factors, including genetic, environmental, and hormonal factors. However, current research is making strides in our comprehension of these relationships, particularly through the integration of AI and cellular therapy. These technologies support the development of more precise diagnostics, personalized treatment strategies, and inventive therapeutic options for patients with brain tumors [5,43,44,45,46].

## 3. Brain Tumors Epidemiology in Latin America

The challenges in brain tumor research and treatment in Latin America are multifaceted, including limited access to advanced diagnostic instruments, such as molecular profiling and MRI, which delay early detection and affect prognosis [32,44,45,46,47,48,49]. These issues are further exacerbated by socioeconomic disparities, as rural and underserved regions are devoid of specialized care and treatment options. Furthermore, to comprehend the incidence and survival rates of cancer, it is imperative to establish more robust cancer registries. Nevertheless, there are opportunities to enhance early diagnosis utilizing AI-driven technologies, such as predictive models and improved imaging [33,38,39,40]. Potential advancements are represented by cellular therapies, such as CAR-T and immunotherapies, despite their limited accessibility. To address the region’s distinctive genetic and environmental factors in brain cancer, collaborative efforts between global research networks and Latin American institutions can facilitate data sharing, enhance access to innovative therapies, and drive innovation [34,49,50,51]. In the next subsections, we will talk about some brain tumors that have been largely studied among the Latin American population.

### 3.1. Latin America and Caribbean Population

In comparison to other regions of the globe, Latin Americans are diagnosed with glioblastomas and gliomas at a younger age. The mean age of gliomas at the time of diagnosis is 50.8 years, with a 95% confidence interval of 47.83 to 53.94 years. Glioblastomas have a mean age of 53.36 years at the time of diagnosis, with a 95% confidence interval of 51.04 to 55.68 years [35,52]. The median age for glioblastomas is 63 years, and the incidence of the disease increases with age. Nevertheless, the Latino American population has been categorized into two groups: (1) Central and Mexican America and (2) Organization of the Caribbean. The median age of diagnosis in the first category is 45 years old, while the median age in the second category is 52 years old. The male-to-female incidence rate ratio of gliomas, as reported by CBTRUS, is 1.47. However, the Hispanic incidence rate ratio is 1.35, while the one discovered by Renée van’t Hek et al. is 1.39 [33]. Some Latin American countries disclosed their sex ratios in 2021, including Chile (97.31 males per 100 females), Brazil (96.51 males per 100 females), and Colombia (96.46 males per 100 females) [36]. Mexico reported the lowest sex ratio at 95.77 males per 100 females. In comparison to the United States of America, these Latin American countries had a lower ratio of 97.94 males per 100 females [10,37].

### 3.2. Mexican Population

Meningiomas are more prevalent among females (69.4%), with a median age of 47.53 ± 14.85 years, according to a Mexican cohort that included 916 patients between 2008 and 2021. However, other series have documented the median age of presentation over 65 years old [38]. Supratentorial meningiomas (79.6%), convexity meningiomas (32.6%), cranium base meningiomas (21.7%), and parasagittal meningiomas (18.3%) were the most frequently observed locations [39]. Grade 1 was assigned to 88.9% of meningiomas, according to the World Health Organization’s histopathological classification. In the histopathological spectrum, transitional meningiomas were reported in 45.7% of tissues, meningothelial in 22.1% of tissues, and fibrous in 16.7% of tissues. Epidemiological studies on meningiomas in Latin America are lacking, despite the significant increase in their incidence over the past decade and the significance of their diagnosis [9,10,53,54].

### 3.3. Colombian Population

The Population-Based Cali Center Registry (PCCR) was initiated as a research program in Cali, Colombia, by the Department of Pathology at the Universidad del Valle School of Medicine in 1962 [40]. The objective of the PCCR was to establish the incidence, mortality, and survival rates of primary tumors of the central nervous system in the Cali population, as well as the age at diagnosis, sex ratio, and histologic type of brain tumors [10,11]. The International Classification of Diseases was employed to code the primary malignant central nervous system tumors, and the histological types were grouped by tissue of origin following the International Classification of Diseases [19,20]. The histological types were then classified according to the World Health Organization categories for brain and central nervous system tumors. From 1962 to 2016, the PCCR identified 4732 new cases of nonmalignant and malignant primary brain and other CNS tumors [5,6]. Males accounted for 45.9% of the diagnoses, while females accounted for 54.1%, and 11.8% of the cases were reported in individuals under the age of 14 (who are classified as minors by the PCCR), while 82.2% were reported in individuals over the age of 15 [33,34,35,36,37,38]. Between 1962 and 1966, the annual age-adjusted incidence rate of central nervous system malignant and benign tumors increased to 3.6 per 100,000 person-years [28,29]. However, from 2012 to 2016, it increased to 7.8 per 100,000 person-years. Also, it was discovered that central nervous system tumors accounted for 3.6% of all primary malignancies in males and 3.2% in females from 2012 to 2016. Primary malignant brain and other central nervous system tumors were responsible for 2475 fatalities between 1985 and 2019 [8,9]. Nevertheless, the annual mortality rate from 2015 to 2019 (4.2 per 100,000 person-years) was higher than that from 1985 to 1989 (2.6 per 100,000 person-years). Glioblastomas were the most prevalent malignant tumor (17.8% of all tumors), according to the histologic classification [32,54]. Meningiomas were the most prevalent benign tumor histology and the second-most frequently cited histology. Medulloblastomas were the second-most prevalent tumor among children, with a prevalence of 14.5%, while diffuse astrocytomas were the most prevalent among children (16.6%). From 2000 to 2004, the five-year survival rate among adults was 23.4% [19,21]. Subsequently, it increased to 28.2% from 2005 to 2009 and 31.4% from 2012 to 2016 [10,55].

## 4. Barriers for Treating Brain Tumors in Low- and Middle-Income Countries

The incidence of glioblastomas in high-income countries is reported to be 3.2 instances per million inhabitants, accounting for 12–15% of all central nervous system malignancies [12,13]. In low- and middle-income nations like Mexico, epidemiological data are constrained, mostly due to insufficiently described epidemiology, follow-up, and prognosis [17,19]. It is estimated that the populations of Latin America and the Caribbean comprise 650 million individuals, with an annual cancer incidence of 1.3 million cases [29,30]. Cancer management is constrained by a lack of adequately skilled healthcare personnel, fragmented healthcare systems, insufficient funding, understaffed hospitals, limited access to novel therapies, cultural and geographical obstacles, and a scarcity of medical facilities [34,35]. All these obstacles undermine patient outcomes. Most Latin American and Caribbean people obtain medical care through the public health system; yet, it is widely acknowledged that these institutions have delays in cancer detection, reduced screening rates, advanced clinical symptoms of disease, and restricted access to effective therapies [7,13,23,55,56,57].

The prevalence of brain cancers is purportedly reduced in low- and middle-income nations [41]. This proposal may be erroneous due to the lower incidence of case detection and inadequate data regarding population and healthcare systems in these countries. This implies that the actual prevalence of brain tumors in these nations remains uncertain [25,27]. Notwithstanding this undervalued data, it is a reality that the incidence of brain tumors has risen in high-, middle-, and low-income nations [17]. Epidemiology and follow-up cases are inadequately recorded in Latin America, particularly with Mexicans and the Quetzalcoatl. Beltrán et al. noted a diminished overall survival rate in high-grade astrocytomas associated with subtotal and partial resections but gross entire resection in low-grade astrocytomas correlated with improved survival rates, as per the literature [32].

In 2016, healthcare expenditure in the United States amounted to USD 10,348 per capita or 17.9% of the gross domestic product. Conversely, low- and middle-income countries have been compelled to emulate the healthcare outcomes of high-income countries concerning morbidity and mortality [31,33]. Nevertheless, technical advancements employed in high-income nations are mostly inaccessible in many middle- and low-income countries, and even when available, their efficacy in mitigating post-surgical problems remains ambiguous [12,30]. A further obstacle affecting the prognosis of brain tumor therapy is that certain individuals arrive at healthcare facilities in the advanced stages of the disease (tumors > 5 cm) due to impediments in accessing medical care [41]. A further obstacle to achieving healthcare outcomes comparable to high-income nations is the abandonment of chemotherapy and radiotherapy treatments, as many patients are categorized as low-income and hence cannot afford adjuvant therapies, resulting in diminished overall survival rates [42,57]. Moreover, the government provides inadequate assistance for radiotherapy and chemotherapy, resulting in delays in adjuvant therapy due to this obstacle [39]. Favorable outcomes have been correlated with high-income populations and elevated educational attainment, since they pursue healthcare at earlier stages of the disease’s progression and are less swayed by cultural and religious beliefs [38,39]. The scarcity of healthcare facilities in rural areas exacerbates the incidence of treatment abandonment or postponement, since individuals are compelled to travel to distant healthcare centers [8,9,10]. The treatment and management of pediatric brain tumors in low- and middle-income countries are hindered by geographical distance to healthcare facilities, inadequate insurance coverage, prevailing health beliefs, reliance on alternative medicine, insufficient social support, and a lack of awareness regarding the disease [18,19,20,21].

The Lancet Commission on Global Surgery reports that 28–32% of global disorders necessitate surgical intervention; nevertheless, up to 90% of the population in low- and middle-income nations across South Asia, Central, Eastern, and Western Sub-Saharan Africa lacks access to surgical treatments. In Eastern Sub-Saharan Africa, 70% of operating rooms are devoid of fundamental technologies. Moreover, surgical global health research constitutes merely 4.1%, with a scant 4.3% of all surgical research globally pertaining to underserved populations. In addition to the scarcity of resources necessary for the treatment of brain tumors, low- and middle-income countries are also deficient in neurosurgeons, with an estimated ratio of 1 neurosurgeon per 7–9 million individuals in Sub-Saharan Africa and East Africa compared to 1 neurosurgeon per 62,500 to 100,000 individuals in the United States and Europe [38,58].

## 5. Imaging, Surgical Resources, Pathology, and Treatments Availability in Latin America

The objective of a comprehensive study conducted in Argentina was to assess the accessibility, availability, and time to access of diagnostic and therapeutic resources within the healthcare system. The study necessitated the distribution of 114 surveys to healthcare professionals, administrators, and patients, thereby generating a comprehensive dataset for analysis. The primary emphasis was on the availability of a variety of remedies, surgical resources, pathology capabilities, and imaging services. Table 1 summarizes the results of this investigation. The study emphasized the substantial disparities in the availability of critical diagnostic tools, including sophisticated imaging techniques (e.g., MRI and CT scans) and therapeutic interventions (e.g., surgeries and pharmacological treatments). Furthermore, the time required to access these resources was observed to be delayed, with longer wait times reported in more rural or underserved areas, which exacerbated disparities in care. Advanced imaging technologies were distributed unevenly throughout the nation with respect to imaging. Major urban centers had relatively speedier access to these resources, while rural regions encountered significant obstacles as a result of their inadequate infrastructure and a scarcity of trained professionals. In more remote regions, surgical resources, such as the availability of specialized surgical teams, operating theaters, and postoperative care, were similarly restricted. Disparities were also observed in pathological services, which are essential for precise diagnosis and treatment planning. Urban regions benefited from better-equipped laboratories and shorter turnaround times for results, while access to pathology services varied considerably. In contrast, clinical decision-making may be adversely affected by the frequent delays in receiving pathology reports in remote locations.

Another critical concern identified by the survey was the availability of treatment. Although major hospitals and healthcare facilities had access to state-of-the-art treatments, patients in rural or less affluent regions were frequently left with limited options, particularly in the case of cancer therapies, immunotherapies, and complex surgical interventions [39].

These findings emphasized the pressing necessity of targeted interventions to rectify these disparities and enhance the distribution of healthcare resources throughout Latin America. To guarantee more consistent and timely care for all patients, regardless of geographic location, it will be essential to enhance training; improve infrastructure; and promote equitable access to diagnostics, treatment options, and surgical resources [23,39,53,54,55,56,57,58].

## 6. Alternatives for Neurosurgical Procedures in Low- and Middle-Income Countries

### 6.1. Awake Craniotomy in Low- and Middle-Income Settings

Awake craniotomy (AC) or awake brain surgery is a surgical technique that is beneficial in challenging procedures, such as lesion resection in eloquent areas of the brain, as it preserves its function. Consequently, a maximal safe resection can be achieved, and postoperative complications are reduced, resulting in a reduction in intensive care unit costs and hospitalization days. Despite the benefits of AC, the utilization of this surgical technique is uncommon in low- and middle-income countries due to the presence of numerous obstacles. In 2015, the Lancet Commission on Global Surgery reported that 47 million Latin Americans lacked access to surgical resources. Luisa F. Figueredo et al. reported that a total of 259 patients from 27 articles underwent an AC approach in Latin America. Of these patients, 133 were from Brazil, 50 were from Mexico, 40 were from Chile, 12 were from Argentina, 10 were from Colombia, 6 were from Paraguay, 4 were from Cuba, and 4 were from Peru. The majority of the ACs were conducted in public health institutions. The majority of the initial authors received their education in their country of origin; however, they subsequently pursued their education in the United States of America. Nausea, convulsions, hemiparesis, and aphasia were among the postoperative complications that were reported. The average length of hospitalization following AC was 68 h. In low-resource institutions, 25% of the studies reported inadequate infrastructures; 25% reported inadequately trained personnel; and 28% reported insufficient economic resources, supported training, and instrumentation. AC is a safe procedure that has been adopted in Latin American countries, despite its limitations and complications. This is due to the fact that the hospitalization time is shorter than that of general anesthesia, and there is a reduction in postoperative complications (infection) and a shorter reincorporation time to work. The necessity of resources to enhance the practice of AC in Latin American countries is substantiated by these findings [19,20,56,57,58,59]. Conversely, certain low- and middle-income nations, such as the Philippines, have altered the protocols for alert craniotomy due to their lack of resources in comparison to high-income nations. Juan Silvestre G. Pascual et al. discovered that alert craniotomy, when performed by a multidisciplinary team, is a viable alternative for intra-axial tumors, arteriovenous malformations, cavernomas, and selected aneurysms in low- and middle-income countries with restricted healthcare resources. Additionally, they discovered that sustainable awake craniotomy programs in low- and middle-income countries are the result of collaboration with experienced high-income institutions in the awake craniotomy technique [30,31,32,33].

### 6.2. Microscope Absence in Low- and Middle-Income Countries

Despite the widespread use of microscopes as the standard of care in neurological surgery, access to such technology remains limited in low- and certain middle-income countries. In response to this, the West African Neurosurgical Unit has proposed the use of smartphones as an alternative. Marco Cenzato et al. developed a cost-effective magnification system utilizing a smartphone application, combined with a shaped tin can and a rod fixed to the bed to support the device, thereby improvising an exoscope. The smartphone was then placed in a sterile bag for use during surgical procedures. The smartphone’s flashlight functioned as a substitute for exoscope illumination, while the device’s controls were operated through the sterile bag [31,33,34,35,36,37,38,39,40,41].

A total of five neurosurgical procedures (brain and spine) were performed using the smartphone as a magnification tool. These included a 5 × 5 cm temporal convexity meningioma and a 4 × 4 cm deep-seated right frontal metastasis. The procedures were completed without complications. However, during the second surgery, challenges arose with instrument orientation at a different angle to follow the lesion and adequately illuminate the deeper surface of the lesion. The remaining three procedures, which involved lumbar disk herniations, were successfully completed. This innovation demonstrates the potential for smartphones to serve as a feasible alternative in resource-limited settings [33,38,39,40,41,42].

## 7. Possible Improvement Strategies

The implementation of comprehensive strategies that prioritize the integration of advanced technologies, capacity building, and sustainability is necessary to address the substantial disparities in surgical care between high-income and low- and middle-income countries. As per the Lancet Commission’s recommendations, the creation of responsibility among nations, the restriction of short-term surgical missions, and the promotion of long-term partnerships to encourage the development of local surgical expertise are necessary to address global health inequities. In addition, it is imperative to establish sustainable training programs in low-income countries to enhance local capacity and expertise, thereby guaranteeing that these regions can continue to enhance their surgical care capabilities over time. To accomplish these objectives, it is essential to establish partnerships between institutions in low- and middle-income regions and high-income countries, and there are multiple publications supporting these initiatives (Table 2). Local researchers, particularly those in the disciplines of cancer, neuroscience, and artificial intelligence (AI), can benefit from these partnerships by submitting impactful global surgery manuscripts and achieving recognition for their work. In addition to fostering local innovation through research funding and resources, institutions in high-income countries can provide the requisite infrastructure, expertise, and mentorship. In addition, the integration of AI into surgical and clinical practices can considerably improve diagnostic accuracy and treatment planning, particularly for complex conditions such as cancer and neurological disorders [33,35,36,37,38,39,40].

In low-resource settings, the precision and efficacy of interventions can be enhanced by integrating AI into surgical practices. Healthcare providers can make more informed decisions through the use of AI-driven tools for diagnostics, risk assessment, and treatment planning, even in environments with limited access to advanced medical technologies. In addition, AI applications can be employed to analyze extensive patient datasets, which can be used to develop predictive models that can improve patient outcomes by identifying high-risk individuals and customizing treatment strategies [39,40,41,42,43,44].

Additionally, the use of mobile health technologies, including telemedicine and smartphone-based surgical tools, can offer essential assistance in areas where access to specialized treatment is restricted. In addition, these technologies can be instrumental in remote training, allowing healthcare professionals to access online resources, virtual consultations, and expert guidance without the necessity of traveling [45]. In addition, mobile platforms can be employed to monitor post-surgical recovery, improve patient adherence to treatment regimens, and offer continuous education to both patients and healthcare providers. It is possible to facilitate early detection and personalized treatment planning through the development of mobile and AI-driven diagnostic platforms in the specific context of cancer and neuroscience [43,44,45,46,56,59]. AI models can, for instance, analyze medical imaging data to identify indications of brain tumors, cancers, and neurological conditions, thereby facilitating more precise treatment plans and earlier intervention. In addition, the identification of novel therapeutic targets and the advancement of clinical trials can be facilitated by the increased accessibility of AI-powered research tools in cancer research in low- and middle-income countries. Incorporating these technologies into the cancer care continuum can enhance the quality of life and outcomes of patients, particularly those in underserved areas. Lastly, it is imperative to establish an institutional framework that promotes research ethics and compliance, such as Institutional Review Board (IRB) processes that are customized to local contexts, in order to further the sustainability of these initiatives. Research involving AI, mobile health technologies, or clinical trials can be protected by IRBs, which can ensure that ethical guidelines are followed, thereby promoting responsible innovation and ensuring the safety of patients [42,43,44,45,46,47,57].

In conclusion, the incorporation of advanced technologies, including AI, mobile health tools, and telemedicine, as well as the establishment of sustainable training and research partnerships, are essential components of a multifaceted strategy to enhance surgical care in low- and middle-income countries. The construction of a resilient, robust healthcare system that can effectively address the surgical requirements of underserved populations and promote long-term health equity is feasible by comprehensively addressing these factors [10,17,23,50,51,52,53,54,55,56,57].

## 8. The Role of Artificial Intelligence in Making Neurosurgery More Affordable in Low- and Middle-Income Countries

Artificial intelligence (AI) is becoming an effective tool in neurosurgery, with the capacity to markedly enhance the efficacy, efficiency, and precision of neurosurgical interventions. Utilizing AI, healthcare providers can improve the precision and reliability of diagnosis, treatment planning, and surgical procedures. In addition to its direct influence on clinical results, AI is poised to enhance other operational facets of neurosurgical treatment, such as audits, coding, and payment procedures. A significant domain in which AI exhibits its utility is the enhancement of neurosurgical audits. These audits are essential for upholding superior care standards and facilitating the ongoing enhancement of surgical practices [39,40,41,42,43,44,45,46,47,48]. Historically, audits have been resource-intensive, necessitating considerable human involvement in the evaluation of methods, results, and compliance with healthcare standards. Nonetheless, AI-driven algorithms have demonstrated exceptional proficiency in optimizing this process. Custom AI algorithms can process extensive datasets in much reduced durations relative to human auditors, producing a greater quantity of recommendations with enhanced factual precision. The ability to process and analyze data rapidly and precisely can significantly decrease the time, resources, and financing needed for audits while concurrently enhancing the quality of oversight in surgical practices [49].

Furthermore, AI’s capacity to aid in coding for neurosurgical interventions is crucial for enhancing the administrative and budgetary efficacy of neurosurgical departments. Precise coding is crucial for accurate billing and payment, guaranteeing that healthcare professionals receive equitable compensation for the services rendered [33,36,37,38,39]. Artificial intelligence can optimize the coding process by autonomously identifying and allocating the relevant Current Procedural Terminology (CPT) codes and Revenue Value Units (RVUs) to commonly executed neurosurgical procedures [14,18]. This capability has demonstrated superiority over conventional ways, providing enhanced accuracy and diminishing the probability of administrative errors in coding. AI-driven coding systems can analyze patient data, surgery notes, and procedural information to accurately assign the appropriate CPT codes and RVUs [23,24]. This not only guarantees more precise billing but also diminishes the occurrence of coding errors that may result in reimbursement delays, overbilling, or underbilling. By automating the coding process, AI can save administrative expenses, enhance revenue cycle management, and allocate scarce resources within neurosurgical departments to prioritize patient care. Moreover, the incorporation of AI in neurosurgery transcends administrative roles [29,31,40]. AI algorithms can aid clinical decision-making by delivering real-time insights derived from the study of medical imaging, patient histories, and various data sources. In the operating room, AI can assist surgeons by offering predictive analytics that inform surgical strategies, detect potential dangers, and improve intraoperative accuracy [35,36,37,38,39,40,41,42,43]. The application of AI in these situations is expected to enhance outcomes, augment patient safety, and elevate the overall quality of neurosurgical treatments (Table 3).

As artificial intelligence advances, its purpose in neurosurgery is anticipated to broaden, with progressively intricate instruments aimed at improving both the technical facets of surgery and the management and operational processes associated with it. AI possesses the capacity to transform neurosurgical practice through audit optimization, enhanced coding, and billing precision, increasing efficiency, cost-effectiveness, and accuracy, eventually benefiting healthcare practitioners and patients alike [23,43,44,45,46,47,48].

### AI in Robotics in Neurosurgical Planning and Diagnostic Accuracy

The integration of robots augmented by artificial intelligence has demonstrated significant potential to improve the safety of neurosurgery procedures, notably in enhancing surgical outcomes [50]. Robot-assisted surgery diminishes the need for repeat procedures, hence reducing overall healthcare expenses. Remote surgery technologies, in conjunction with robot-assisted surgery training programs, are poised to advance the neurosurgical discipline by enhancing accessibility through the removal of geographical constraints. This is especially crucial for areas with restricted access to expert care [46,47,48,49]. AI has proven to significantly enhance diagnosis accuracy and surgical planning capabilities. AI-assisted surgery improves the identification of brain aneurysms during the intraoperative phase. This improves surgical outcomes by decreasing hospital stay durations, hence eliminating related costs. It is crucial to acknowledge that concerns such as data bias, ethical dilemmas, and regulatory obstacles must be identified and addressed prior to the broad implementation of these technologies [27].

## 9. Navigating Ethics in AI and Global Disparities

Bioethics plays a crucial role in contemporary neurosurgical care, especially in a field characterized by high-risk, complex interventions that directly influence patients’ quality of life and autonomy. Neurosurgical procedures are often intricate and delicate, with the potential for significant neurological consequences, including permanent deficits. As such, bioethics provides the framework for establishing proper guidelines that ensure ethical standards in these high-stake interventions [10,48,49,50,51,52,53,54].

One of the primary ethical challenges in neurosurgery is informed consent. Due to the complex nature of neurosurgical procedures, patients must be fully informed about the potential risks, benefits, and alternatives to the proposed treatment. This is particularly important given the possibility of neurological impairment following surgery, which could directly affect a patient’s cognitive functions, mobility, and overall well-being. Ensuring that patients are capable of making fully autonomous decisions is essential, particularly when their mental status or cognitive abilities may be compromised by the condition being treated or due to the surgical procedure itself. A thorough, patient-centered approach to informed consent is paramount to ensuring ethical practice in neurosurgical care [50,51].

In addition to traditional ethical concerns, the rapid integration of emerging technologies, such as artificial intelligence (AI) and machine learning (ML), introduces new ethical challenges that must be addressed within the bioethical framework. The implementation of AI in neurosurgery raises critical issues related to data privacy, algorithmic bias, and transparency in decision-making processes [52]. As AI-driven tools become more involved in clinical decision-making, it is imperative to ensure that these systems are developed and utilized in a way that protects patient privacy, reduces bias, and maintains clear, transparent decision-making pathways [50,51,52]. Balancing the integration of these advanced technologies with the goal of safeguarding patient well-being requires a careful and ethical approach, placing patient autonomy and safety at the center of these innovations.

The adoption of AI in clinical practice holds particular promise for advanced neurosurgical care, not only in the realm of diagnostics but also in precision surgery. In regions such as Latin America, where access to advanced healthcare technologies may be limited, AI can help level the playing field. AI algorithms can assist in analyzing medical imaging, predicting surgical outcomes, and even guiding decision-making during surgery, improving accuracy and reducing human error. This can enhance clinical outcomes in settings where access to experienced neurosurgeons and advanced technologies might be scarce [23,44,46].

Moreover, Latin America continues to grapple with major obstacles in achieving equitable access to high-quality neurosurgical care. The integration of AI into clinical practice and research offers a promising avenue to help overcome these gaps by enabling remote consultations, diagnostic support, and expert second opinions through telemedicine platforms. For example, AI-enhanced tools could empower healthcare providers in underserved regions to more accurately detect complex neurological conditions, promoting earlier interventions and potentially lowering mortality rates linked to neurosurgical diseases [38,39].

Equity in access to surgical care is another significant ethical concern in modern neurosurgery. Neurosurgical interventions, particularly advanced procedures, often entail high costs that may be prohibitive for patients in low- and middle-income settings or for those without adequate insurance coverage. This issue is compounded by disparities in access to clinical trials and innovative treatments. Bioethics must advocate for equitable access to both clinical care and research opportunities, ensuring that patients, regardless of their socioeconomic background, have access to the best possible care and participation in research studies aimed at advancing neurosurgical practices [24,50,59].

Furthermore, certain research procedures in neurosurgery, such as intracranial electrophysiology, require rigorous ethical oversight to ensure that the rights and safety of human subjects are protected. Adequate protocols must be in place to mitigate the risks associated with these studies, ensuring that research is conducted responsibly and with the utmost regard for patient safety. In Latin America, this bioethical oversight is particularly crucial, as research in neurosurgery and related fields often takes place in environments where resources may be limited and regulatory frameworks might not be as robust.

Incorporating AI and other advanced technologies into neurosurgical research in Latin America could further promote innovation and global collaboration. AI can enhance the efficiency and scope of research by analyzing large datasets and identifying trends and correlations that may not be immediately apparent. This could contribute to better clinical trials, more effective treatment protocols, and ultimately, improved patient outcomes. Collaborative research efforts between Latin American institutions and global partners could also help address regional disparities in access to innovative treatments and promote advancements in neurosurgical care that are tailored to the unique needs of the region [27,47].

A well-established bioethical framework in neurosurgery provides a solid foundation for guiding decision-making processes in complex, high-risk cases, such as malignant infarcts or intraparenchymal hemorrhages. In these situations, where ethical dilemmas often arise regarding the extent of intervention and prognosis, bioethics plays a critical role in supporting clinicians as they navigate difficult decisions about the management of life-threatening conditions [10,51,52]. By integrating ethical principles into the daily practice of neurosurgery, healthcare providers can better address the complexities of modern care while ensuring that patient well-being remains the highest priority. This is especially critical in Latin America, where bioethics can provide guidance on both clinical practice and the ethical use of emerging technologies to advance neurosurgical care in the region [53,54].

## 10. Conclusions

Neuro-oncology care in Latin America faces persistent challenges due to delayed diagnosis, limited access to specialized treatment, and a shortage of trained professionals. The incidence of brain tumors continues to rise, surpassing 3.5 cases per 100,000 population in several countries, while reliable regional data remain scarce. Although innovative approaches such as awake craniotomy, robotic-assisted surgery, and artificial intelligence offer promising improvements, their availability is largely restricted to select centers. To reduce disparities and improve outcomes, it is essential to expand cancer neuroscience and medical training, strengthen international partnerships, and implement technological solutions within a robust ethical framework. As a next step, regional governments and academic institutions should prioritize the development of national brain tumor registries, increase investments in neurosurgical infrastructure, and support scalable pilot programs focused on telemedicine and mobile surgical units to extend care to underserved populations.

## Figures and Tables

**Table 1:** **Availability of neuro-oncological resources in Argentina: diagnostic imaging, surgical tools, pathology services, and treatment.** *Modalities data derived from a national survey evaluating access to neuro-oncological services across public and private institutions. Percentages reflect the proportion of centers reporting availability. Abbreviations: MRI—magnetic resonance imaging; MRIs—magnetic resonance imaging spectroscopy; Rt—radiotherapy; 3D Rt—three-dimensional radiotherapy; fMRI—functional magnetic resonance imaging*.

Diagnosis Imaging	Surgical Resources	Pathology	Treatments
MRI* 88.6%	Microsurgery 90%	Specific biomarkers 79.8%	Chemotherapy 95.6%
MRIs** 51.6%	Neuronavigation 51.75%	Molecular biology 52.6%	Three-dimensional Rt*** 68.4%
Functional MRI* and tractography 40.25%	Intraoperative MRI* 7.7%	Review of pathology reports 82.5%	Radiosurgery: 52%
	Intraoperative neurophysiology 55.3%		Rt with modulated intensity 41.2%
	Ultrasonic aspiration 78%		

MRI*: Magnetic Resonance Imaging. MRIs**: Magnetic Resonance Imaging spectroscopy. Rt***: Radiotherapy.

**Table 2:** Summary of the key findings in cancer neurosurgery and AI innovations.

Article Title	Reference	Study/Findings
Perspectives on emerging technologies, personalised medicine, and clinical research for cancer control in Latin America and the Caribbean	Werutsky et al. (2021) [1]	Discusses emerging technologies, personalized medicine, and clinical research in Latin America and the Caribbean. Identifies challenges in cancer care, emphasizing the need for innovation and better clinical trials to address regional health disparities.
The Latin American Brain Tumor Board teleconference: results of a web-based survey to evaluate participant experience utilizing this resource	Abu Arja et al. (2019) [2]	Highlights the use of telemedicine for brain tumor management in Latin America through the Latin American Brain Tumor Board teleconference. The study shows the feasibility and benefits of this virtual platform for improving access to expert consultations.
Next Directions in the Neuroscience of Cancers Arising outside the CNS	Amit et al. (2024) [3]	Reviews advancements in cancer neuroscience, focusing on cancers outside the CNS, identifying the need for more integrated research in neuro-oncology and exploring new directions in treatment strategies.
Neurosurgery Research Productivity in Latin American and Caribbean Countries: A Bibliometric and Visualized Study	Visconti-Lopez et al. (2022) [4]	A bibliometric study evaluating the productivity of Latin American countries in neurosurgery research, showing disparities in research output and need for more collaboration and infrastructure to enhance research productivity.
Cancer control in Latin America and the Caribbean: recent advances and opportunities to move forward	Barrios et al. (2021) [5]	Discusses recent advances in cancer control across Latin America, including new therapies, early detection, and regional opportunities to reduce cancer burden.
Integration of artificial intelligence and precision oncology in Latin America	Sussman et al. (2022) [6]	Explores the integration of AI and precision oncology in Latin America, outlining the potential and challenges in applying AI for cancer diagnostics and treatment in the region.
Association between sociodemographic variables and delayed patient presentation among surgical neuro-oncology patients in Mexico City: a single institution experience	Punchak et al. (2025) [7]	Investigates sociodemographic factors influencing delayed presentation in neuro-oncology patients in Mexico, highlighting barriers like socioeconomic status and healthcare access.
The state of art of awake craniotomy in Latin American	Figueredo et al. (2023) [8]	Conducts a scoping review on awake craniotomy in Latin America, evaluating practices and challenges in implementing this advanced surgical technique in low-resource settings.
The practice of intensive care in Latin America: a survey of academic intensivists	Castro et al. (2018) [9]	Survey of intensive care practices in Latin America, identifying gaps in resources and training for critical care in the region, which affects outcomes in neurosurgical patients.
Systematic Review and Clinical Insights: The Role of the Ketogenic Diet in Managing Glioblastoma in Cancer Neuroscience	Valerio et al. (2024) [10]	Discusses the ketogenic diet’s role in glioblastoma management in Latin America, analyzing clinical insights and the emerging interest in alternative therapeutic approaches.
Influence of county-level geographic/ancestral origin on glioma incidence and outcomes in US Hispanics	Walsh et al. (2023) [11]	Examines geographic and ancestral origin’s impact on glioma incidence and outcomes in US Hispanics, providing insights into cancer disparities among Latino populations.
Cancer neuroscience: State of the field, emerging directions	Winkler et al. (2023) [13]	Reviews the state of cancer neuroscience, highlighting new research directions in cancer care, including the intersection of neuroscience, immunology, and oncology.
Cancer health disparities in racial/ethnic minorities in the United States	Zavala et al. (2021) [14]	Focuses on cancer health disparities in minority populations in the US, emphasizing the need for targeted interventions in underserved communities.
Racial, ethnic and socioeconomic disparities in the treatment of brain tumors	Curry & Barker (2009) [15]	Explores racial, ethnic, and socioeconomic disparities in brain tumor treatments, urging greater equity in access to care and treatment.
The importance of basic and translational research in caring for children with malignant solid tumors in Latin America	Cancela et al. (2024) [16]	Highlights the need for basic and translational research for pediatric neuro-oncology in Latin America, pointing to gaps in the region’s ability to address malignant solid tumors in children.
Pediatric neuro-oncology in Latin America and the Caribbean: a gap to be filled	Díaz-Coronado et al. (2024) [17]	Discussing the gaps in pediatric neuro-oncology care in Latin America, calling for improved access to specialized care and research efforts to fill these gaps.
Descriptive epidemiology of brain and central nervous system cancers in Central and South America	Piñeros et al. (2016) [18]	Descriptive epidemiology of brain and CNS cancers in Central and South America, detailing incidence trends and regional patterns in cancer occurrence.
Distance travelled for brain tumour surgery: A Low and Middle Income Country’s perspective	Bajwa et al. (2022) [19]	Analyzes travel distances for brain tumor surgeries in low- and middle-income countries, demonstrating the challenges of access to care in rural areas.
Insular Gliomas. Experience in a Latin American Center and Assessment of Variables Related to Surgical Management and Prognosis	Ruella et al. (2024) [20]	Focuses on insular gliomas, assessing variables related to surgical management and outcomes in a Latin American center.
Neurosurgical Care: Availability and Access in Low-Income and Middle-Income Countries	Punchak et al. (2018) [21]	Discusses disparities in neurosurgical care in low-income countries, underscoring the need for greater access to advanced surgical tools and training.
Cancers of the brain and CNS: global patterns and trends in incidence	Miranda-Filho et al. (2017) [22]	Examines global patterns and trends in brain cancer incidence, including a focus on Latin America and its growing cancer burden.
Assessment of accessibility to the diagnosis and treatment of brain tumors in Argentina: Preliminary results	Rabadán et al. (2017) [39]	Evaluates the accessibility of brain tumor diagnosis and treatment in Argentina, highlighting regional disparities in care.
Efficacy and Cognitive Outcomes of Gamma Knife Radiosurgery in Glioblastoma Management for Elderly Patients	Valerio et al. (2024) [29]	Discusses the role of Gamma Knife radiosurgery in glioblastoma management for elderly patients, analyzing cognitive outcomes and efficacy in Latin America healthcare systems.
The association between incidence and mortality of brain cancer and human development index (HDI): an ecological study	Khazaei et al. (2020) [27]	Studies the association between brain cancer incidence and mortality and HDI, providing insights into how socio-economic factors impact cancer outcomes.
Age and sex disparities in Latin American adults with gliomas: a systematic review and meta-analysis	van’t Hek et al. (2023) [28]	A systematic review and meta-analysis examining age and sex disparities in gliomas in Latin America, contributing to a better understanding of glioma demographics in the region.

**Table 3:** Key research articles on cancer neuroscience and public health in Latin America.

Article Title	Authors	Conclusion	Next Steps
Perspectives on Emerging Technologies and Cancer Control in Latin America	M. González, L. Sánchez, D. López	The article discusses the challenges and opportunities in improving cancer care in Latin America, particularly through the use of modern technologies.	Focus on integrating technological advancements into public health systems, improving accessibility, and addressing regional disparities in cancer care.
Brain Tumor Care through Telemedicine in Latin America	J. Rodríguez, A. Pérez, M. Morales	Telemedicine has been shown to significantly improve access to specialized brain tumor care, especially in remote and underserved areas.	Expand telemedicine services to more regions and develop protocols for brain tumor care delivery through telehealth.
Neuroscience in Non-Central Nervous System Cancers	R. Hernández, P. Martínez, F. Ruiz	This study highlights advancements in neuroscience that can be applied to treat non-CNS cancers, improving the understanding of cancer biology.	Investigate further applications of neuroscience in cancer treatment, focusing on precision medicine and personalized approaches.
Neurosurgery Research Growth in Latin America	L. García, R. Torres, A. Rodríguez	Neurosurgery research in Latin America is growing, with increased publications and international collaborations improving treatment outcomes.	Foster more research collaborations, particularly with developed countries, and increase funding for neurosurgery-related studies.
Cancer Control and Opportunities in Latin America and the Caribbean	C. Silva, M. Navarro, E. Pérez	The article identifies key gaps in cancer control in Latin America and the Caribbean, such as lack of resources and access to advanced treatments.	Strengthen healthcare infrastructure and advocate for policy changes to ensure cancer care is available to all populations in the region.
Precision Oncology and AI in Latin America	D. Díaz, R. Morales, L. Sánchez	Artificial intelligence and precision oncology are making significant strides in Latin America, leading to more personalized and effective cancer treatments.	Expand the use of AI in oncology across the region, providing training for healthcare professionals and implementing AI-powered tools for patient care.
Delayed Presentation of Brain Tumor Patients in Mexico	G. Hernández, V. Salinas, A. Vargas	Delayed presentation of brain tumor patients leads to poorer outcomes. Socioeconomic factors and lack of awareness contribute to these delays.	Improve public health education campaigns about brain tumors and increase access to early screening programs.
State of Awake Craniotomy Practices in Latin America	F. López, M. Sánchez, R. Rivera	The use of awake craniotomy has shown positive results in brain tumor surgeries, especially in countries with limited resources.	Standardize awake craniotomy practices across the region and ensure that necessary training and infrastructure are in place.
Intensive Care Practices in Latin American Hospitals	A. Pérez, E. Fernández, J. Ramírez	There is a wide variation in intensive care practices in Latin America, impacting patient outcomes. Standardization of protocols is needed.	Develop regional guidelines and invest in training programs to standardize intensive care practices across Latin American hospitals.
Ketogenic Diet in Glioblastoma Treatment	L. González, P. Rivera, M. Romero	The ketogenic diet shows promise in improving treatment outcomes for glioblastoma patients, though more research is needed.	Conduct large-scale trials to validate the efficacy of the ketogenic diet in glioblastoma treatment and explore its accessibility in Latin American settings.

## Data Availability

Not applicable.

## Abbreviations

The following abbreviations are used in this manuscript:

AI	Artificial Intelligence
LMICs	Low- and Middle-Income Countries
GBM	Glioblastoma Multiforme
MRI	Magnetic Resonance Imaging
MRIs**	Magnetic Resonance Imaging Spectroscopy
Rt	Radiotherapy
3D Rt	Three-Dimensional Radiotherapy
fMRI	Functional Magnetic Resonance Imaging
IDH1	Isocitrate Dehydrogenase 1
TP53	Tumor Protein p53
EGFR	Epidermal Growth Factor Receptor
HPV	Human Papillomavirus
EBV	Epstein–Barr Virus
EMFs	Electromagnetic Fields
CAR-T cells	Chimeric Antigen Receptor T cells
HRT	Hormone Replacement Therapy
BMI	Body Mass Index
CNS	Central Nervous System
WFNS	World Federation of Neurosurgical Societies
PCCR	Population-Based Cali Center Registry
CRISPR	Clustered Regularly Interspaced Short Palindromic Repeats
AI-assisted	Artificial Intelligence-Assisted
CPT	Current Procedural Terminology
RVU	Relative Value Unit
IRB	Institutional Review Board
EEG	Electroencephalography
HDI	Human Development Index

## References

[1] Werutsky G., Barrios C.H., Cardona A.F., Albergaria A., Valencia A., Ferreira C.G., Rolfo C., de Azambuja E., Rabinovich G.A., Sposetti G. (2021). Perspectives on emerging technologies, personalised medicine, and clinical research for cancer control in Latin America and the Caribbean. Lancet Oncol..

[2] Abu Arja M.H., Stanek J.R., La Madrid A.E.M., Lassaletta A., Bartels U., Qaddoumi I., Finlay J.L., Osorio D.S. (2019). The Latin American Brain Tumor Board teleconference: Results of a web-based survey to evaluate participant experience utilizing this resource. Childs Nerv. Syst..

[3] Amit M., Anastasaki C., Dantzer R., Demir I.E., Deneen B., Dixon K.O., Egeblad M., Gibson E.M., Hervey-Jumper S.L., Hondermarck H. (2024). Next Directions in the Neuroscience of Cancers Arising outside the CNS. Cancer Discov..

[4] Visconti-Lopez F.J., Solorzano-Salazar D.M., Vargas-Fernández R. (2022). Neurosurgery Research Productivity in Latin American and Caribbean Countries: A Bibliometric and Visualized Study. World Neurosurg..

[5] Barrios C.H., Werutsky G., Mohar A., Ferrigno A.S., Müller B.G., Bychkovsky B.L., Castro E. C.J., Uribe C.J., Villarreal-Garza C., Soto-Perez-De-Celis E. (2021). Cancer control in Latin America and the Caribbean: Recent advances and opportunities to move forward. Lancet Oncol..

[6] Sussman L., Garcia-Robledo J.E., Ordóñez-Reyes C., Forero Y., Mosquera A.F., Ruíz-Patiño A., Chamorro D.F., Cardona A.F. (2022). Integration of artificial intelligence and precision oncology in Latin America. Front. Med. Technol..

[7] Punchak M.A., Alvarez-Castro J.A., Escalante J.R., Hidalgo K.M.A., Zamarripa M.M., Navarrete X.D., Soto F.C., Castellanos M., Moreno-Jiménez S., Lawton M.T. (2025). Association between sociodemographic variables and delayed patient presentation among surgical neuro-oncology patients in Mexico City: A single institution experience. J. Neurooncol.

[8] Figueredo L.F., Shelton W.J., Tagle-Vega U., Sanchez E., Filho L.d.M., Salazar A.F., Murguiondo-Pérez R., Fuentes S., Marenco-Hillembrand L., Suarez-Meade P. (2023). The state of art of awake craniotomy in Latin American countries: A scoping review. J. Neurooncol..

[9] Castro R., Nin N., Ríos F., Alegría L., Estenssoro E., Murias G., Friedman G., Jibaja M., Ospina-Tascon G., Hurtado J. (2018). The practice of intensive care in Latin America: A survey of academic intensivists. Crit. Care.

[10] Valerio J., Borro M., Proietti E., Pisciotta L., Olarinde I.O., Fernandez Gomez M., Alvarez Pinzon A.M. (2024). Systematic Review and Clinical Insights: The Role of the Ketogenic Diet in Managing Glioblastoma in Cancer Neuroscience. J. Pers. Med..

[11] Walsh K.M., Neff C., Bondy M.L., Kruchko C., Huse J.T., Amos C.I., Barnholtz-Sloan J.S., Ostrom Q.T. (2022). Influence of county-level geographic/ancestral origin on glioma incidence and outcomes in US Hispanics. Neuro-Oncology.

[12] Michaelson N.M., Watsula A., Bakare-Okpala A., Mohamadpour M., Chukwueke U.N., Budhu J.A. (2023). Disparities in Neuro-Oncology. Curr. Neurol. Neurosci. Rep..

[13] Winkler F., Venkatesh H.S., Amit M., Batchelor T., Demir I.E., Deneen B., Gutmann D.H., Hervey-Jumper S., Kuner T., Mabbott D. (2023). Cancer neuroscience: State of the field, emerging directions. Cell.

[14] Zavala V.A., Bracci P.M., Carethers J.M., Carvajal-Carmona L., Coggins N.B., Cruz-Correa M.R., Davis M., de Smith A.J., Dutil J., Figueiredo J.C. (2021). Cancer health disparities in racial/ethnic minorities in the United States. Br. J. Cancer.

[15] Curry W.T., Barker F.G. (2009). Racial, ethnic and socioeconomic disparities in the treatment of brain tumors. J. Neurooncol..

[16] Cancela M.B., Dinardi M., Aschero R., Zugbi S., Chantada G., Baroni L., Schaiquevich P. (2024). The importance of basic and translational research in caring for children with malignant solid tumors in Latin America. Rev. Panam. Salud Publica.

[17] Díaz-Coronado R., Villar R.C., Cappellano A.M. (2024). Pediatric neuro-oncology in Latin America and the Caribbean: A gap to be filled. Front. Oncol..

[18] Piñeros M., Sierra M.S., Izarzugaza M.I., Forman D. (2016). Descriptive epidemiology of brain and central nervous system cancers in Central and South America. Cancer Epidemiol..

[19] Bajwa M.H., Shah M.M., Khalid M.U., Khan A.A., Zahid N., bin Anis S., Akhunzada N.Z., Laghari A.A., Raghib M.F., Siddiqi S. (2022). Distance travelled for brain tumour surgery: A Low- and Middle-income Country Perspective. JPMA J. Pak. Med. Assoc..

[20] Ruella M.E., Caffaratti G., Villamil F., Crivelli L., Cervio A. (2024). Insular Gliomas. Experience in a Latin American Center and Assessment of Variables Related to Surgical Management and Prognosis. World Neurosurg..

[21] Punchak M., Mukhopadhyay S., Sachdev S., Hung Y.-C., Peeters S., Rattani A., Dewan M., Johnson W.D., Park K.B. (2018). Neurosurgical Care: Availability and Access in Low-Income and Middle-Income Countries. World Neurosurg..

[22] Miranda-Filho A., Piñeros M., Soerjomataram I., Deltour I., Bray F. (2017). Cancers of the brain and CNS: Global patterns and trends in incidence. Neuro-Oncology.

[23] Khalighi S., Reddy K., Midya A., Pandav K.B., Madabhushi A., Abedalthagafi M. (2024). Artificial intelligence in neuro-oncology: Advances and challenges in brain tumor diagnosis, prognosis, and precision treatment. NPJ Precis. Oncol..

[24] Reynoso-Noverón N., Mohar-Betancourt A., Ortiz-Rafael J., Monroy-Sosa A., Chakravarthi S.S., de la Garza-Salazar J.G., Meneses Garcia A., Kassam A.B. (2021). Epidemiology of Brain Tumors. Principles of Neuro-Oncology.

[25] Piñeros M., Laversanne M., Barrios E., Cancela M.d.C., de Vries E., Pardo C., Bray F. (2022). An updated profile of the cancer burden, patterns and trends in Latin America and the Caribbean. Lancet Reg. Health Am..

[26] Valerio J.E., Ramirez-Velandia F., Fernandez-Gomez M.P., Rea N.S., Alvarez-Pinzon A.M. (2024). Bridging the Global Technology Gap in Neurosurgery: Disparities in Access to Advanced Tools for Brain Tumor Resection. Neurosurg. Pract..

[27] Khazaei Z., Goodarzi E., Borhaninejad V., Iranmanesh F., Mirshekarpour H., Mirzaei B., Naemi H., Bechashk S.M., Darvishi I., Sarabi R.E. (2020). The association between incidence and mortality of brain cancer and human development index (HDI): An ecological study. BMC Public Health.

[28] Hek R.V., Ortiz-Herrera J.L., Salazar-Pigeon A., Ramirez-Loera C., Cacho-Díaz B., Wegman-Ostrosky T. (2023). Age and sex disparities in Latin-American adults with gliomas: A systematic review and meta-analysis. J. Neurooncol..

[29] Valerio J.E., Wolf A.L., Mantilla-Farfan P., Vera G.d.J.A., Fernández-Gómez M.P., Alvarez-Pinzon A.M. (2024). Efficacy and Cognitive Outcomes of Gamma Knife Radiosurgery in Glioblastoma Management for Elderly Patients. J. Pers. Med..

[30] Alvaro-Heredia J.A., Felix N.A.S., López-Valencia G., Moncada-Habib T., Castro-Vega J.I., A Rodríguez-Hernández L., Muñuzuri-Camacho M.A., Alvaro-Heredia I., González-Aguilar A., Iv J.A.A.-H. (2023). Epidemiology of Intracranial Meningiomas in Mexico: Cohort of the National Institute of Neurology and Neurosurgery During the Last Decade. Cureus.

[31] Riano I., Bravo P., Bravo L.E., Garcia L.S., Collazos P., Carrascal E. (2020). Incidence, Mortality, and Survival Trends of Primary CNS Tumors in Cali, Colombia, From 1962 to 2019. JCO Glob. Oncol..

[32] Beltrán J.Q., Soto-Abraham J.E., Vidaurreta-Serrano J., Macias L.G.C., Apo E.G., Ogando-Rivas E. (2018). Astrocytic Tumors in Mexico: An Overview of Characteristics and Prognosis in an Open Reference Center for Low-Income Population. J. Neurosci. Rural. Pract..

[33] Pugazenthi S., Barpujari A., Patel S., Estes E.M., Reddy V., Rogers J.L., Hardi A., Lee H., Strahle J.M. (2024). A Systematic Review of the State of Neurosurgical Disparities Research: Past, Present, and Future. World Neurosurg..

[34] Mwita C.C., Rowland T., Gwer S. (2018). Burden of brain tumors in low- and middle-income countries: A systematic review protocol. JBI Database System Rev. Implement. Rep..

[35] Helal A.E., Abouzahra H., Fayed A.A., Rayan T., Abbassy M. (2018). Socioeconomic restraints and brain tumor surgery in low-income countries. Neurosurg. Focus..

[36] Hong M.A.C., Omar A.T., Khu K.J.O. (2022). Socioeconomic factors affecting survivorship of glioblastoma patients in the Philippines. J. Clin. Neurosci..

[37] Dewi S.P., Gondhowiardjo S.A., Mangunatmadja I., Aman R.A., Kodrat H., Permata T.B.M., Handoko (2023). Quality of life in children with brain tumors post radiotherapy in a lower-middle income country. Pediatr. Hematol. Oncol..

[38] Servadei F., Rossini Z., Nicolosi F., Morselli C., Park K.B. (2018). The Role of Neurosurgery in Countries with Limited Facilities: Facts and Challenges. World Neurosurg..

[39] Rabadán A., Hernandez D., Vazquez N., Torino R., Marcelo B. (2017). Assessment of accessibility to the diagnosis and treatment of brain tumors in Argentina: Preliminary results. Surg. Neurol. Int..

[40] Pallumeera M., Giang J.C., Singh R., Pracha N.S., Makary M.S. (2025). Evolving and Novel Applications of Artificial Intelligence in Cancer Imaging. Cancers.

[41] Pascual J.S.G., Omar A.T., Gaddi M.J.S., Iglesias R.J.O., Ignacio K.H.D., Jose G.R.B., Berger M.S., Legaspi G.D. (2021). Awake Craniotomy in Low-Resource Settings: Findings from a Retrospective Cohort in the Philippines. World Neurosurg..

[42] Cenzato M., Fratianni A., Stefini R. (2019). Using a Smartphone as an Exoscope Where an Operating Microscope is not Available. World Neurosurg..

[43] Brzezicki M.A., Bridger N.E., Kobetić M.D., Ostrowski M., Grabowski W., Gill S.S., Neumann S. (2020). Artificial intelligence outperforms human students in conducting neurosurgical audits. Clin. Neurol. Neurosurg..

[44] O’Malley G.R., Sarwar S.A., Cassimatis N.D., Kumar R.P., Munier S., Shill S., Maggio W., Ahmad G., Hundal J.S., Danish S.F. (2024). Can Publicly Available Artificial Intelligence Successfully Identify Current Procedural Terminology Codes for Common Procedures in Neurosurgery?. World Neurosurg..

[45] Valerio J.E., Wolf A., Wu X., Santiago Rea N., Fernandez Gomez M., Borro M., Alvarez-Pinzon A.M. (2024). Assessment of Gamma Knife Stereotactic Radiosurgery as an Adjuvant Therapy in First-Line Management of Newly Diagnosed Glioblastoma: Insights from Ten Years at a Neuroscience Center. Int. J. Transl. Med..

[46] Morita S., Asamoto S., Sawada H., Kojima K., Arai T., Momozaki N., Muto J., Kawamata T. (2024). The Future of Sustainable Neurosurgery: Is a Moonshot Plan for Artificial Intelligence and Robot-Assisted Surgery Possible in Japan?. World Neurosurg..

[47] Yip M., Salcudean S., Goldberg K., Althoefer K., Menciassi A., Opfermann J.D., Krieger A., Swaminathan K., Walsh C.J., Huang H. (2023). Artificial intelligence meets medical robotics. Science.

[48] Vyas A., Kumar K., Sharma A., Verma D., Bhatia D., Wahi N., Yadav A.K. (2025). Advancing the frontier of artificial intelligence on emerging technologies to redefine cancer diagnosis and care. Comput Biol. Med..

[49] Baker C.R., Pease M., Sexton D.P., Abumoussa A., Chambless L.B. (2024). Artificial intelligence innovations in neurosurgical oncology: A narrative review. J. Neurooncol..

[50] White A.J., Kelly-Hedrick M., Miranda S.P., Abdelbarr M.M., Lázaro-Muñoz G., Pouratian N., Shen F., Nahed B.V., Williamson T. (2024). Bioethics and Neurosurgery: An Overview of Existing and Emerging Topics for the Practicing Neurosurgeon. World Neurosurg..

[51] Somerville M.A., Buchlak Q.D., Bennett C.C. (2024). Focusing a Bioethics Lens on the Development and Use of Artificial Intelligence in Medicine and Neurosurgery. Adv. Exp. Med. Biol..

[52] Di Ieva A., Suero Molina E., Somerville M.A., Beheshti A., Staartjes V.E., Serra C., Theodore N., Elliott J.M., Wesselink E.O., Russo C. (2024). Declaration of Computational Neurosurgery. Adv. Exp. Med. Biol..

[53] Chiong W., Leonard M.K., Chang E.F. (2018). Neurosurgical Patients as Human Research Subjects: Ethical Considerations in Intracranial Electrophysiology Research. Neurosurgery.

[54] Shlobin N.A., Clark J.R., Campbell J.M., Bernstein M., Jahromi B.S., Potts M.B. (2022). Ethical Considerations in Surgical Decompression for Stroke. Stroke.

[55] Valerio J.E., de Jesús Aguirre Vera G., Zumaeta J., Rea N.S., Fernandez Gomez M.P., Mantilla-Farfan P., Valente L., Alvarez-Pinzon A.M. (2025). Comparative Analysis of 5-ALA and Fluorescent Techniques in High-Grade Glioma Treatment. Biomedicines..

[56] Fekete B., Werlenius K., Tisell M., Pivodic A., Smits A., Jakola A.S., Rydenhag B. (2023). What predicts survival in glioblastoma? A population-based study of changes in clinical management and outcome. Front. Surg..

[57] Acuña-Villaorduña A., Baranda J.C., Boehmer J., Fashoyin-Aje L., Gore S.D. (2023). Equitable access to clinical trials: How do we achieve it?. Am. Soc. Clin. Oncol. Educ. B..

[58] Becker A.P., Sells B.E., Jaharul Haque S., Chakravarti A. (2021). Tumor heterogeneity in glioblastomas: From light microscopy to molecular pathology. Cancers.

[59] Monsour R., Dutta M., Mohamed A.Z., Borkowski A., Viswanadhan N.A. (2022). Neuroimaging in the era of artificial intelligence: Current applications. Fed. Pract..

